# Staging lymphadenectomy in patients with localized high risk prostate cancer: comparison of the laparoendoscopic single site (LESS) technique with conventional multiport laparoscopy

**DOI:** 10.1186/1471-2490-14-92

**Published:** 2014-11-21

**Authors:** Frank Friedersdorff, Seven Johannes Aghdassi, Ahmed Magheli, Maximilian Richter, Carsten Stephan, Jonas Busch, Dirk Boehmer, Kurt Miller, T Florian Fuller

**Affiliations:** Department of Urology, Charité University Hospital, Charitéplatz 1, 10117 Berlin, Germany; Department of Radiation Oncology, Charité University Hospital, Berlin, Germany

**Keywords:** Laparoscopy, LESS, Single port, Prostate cancer staging

## Abstract

**Background:**

In patients with localized high-risk prostate cancer awaiting radiation therapy, pelvic lymphadenectomy (PL) is a reliable minimally invasive staging procedure. We compared outcomes after laparoendoscopic single site PL (LESSPL) with those after conventional multiport laparoscopic PL (MLPL).

**Methods:**

A retrospective case-control study was carried out at the authors’ center. For LESSPL the reusable X-Cone single port was combined with straight and prebent laparoscopic instruments and an additional 3 mm needlescopic grasper. MLPL was performed via four trocars of different sizes using standard laparoscopic instruments.

**Results:**

Patients who underwent either LESSPL (n = 20) or MLPL (n = 97) between January 2008 and July 2013, were included in the study. Demographic data were comparable between groups. Patients in the LESSPL group tended to be older and had a significantly higher ASA-score. The mean operating time was 172.4 ± 34.1 min for LESSPL and 116.6 ± 40.1 min for MLPL (*P* < .001). During LESSPL, no conversion to MLPL was necessary. An average of 12 lymph nodes per patient was retrieved, with no significant difference between study groups. Postoperative pain scores were similar between groups. The hospital stay was 2.3 ± 0.7 days after LESSPL and 3.1 ± 1.2 days after MLPL (*P* = .01). Two days postoperatively, significantly more patients after LESSPL than after MLPL recovered their normal physical activity (*P* < .001). Six months postoperatively, no complications were registered in the LESSPL group and cosmetic results were excellent.

**Conclusions:**

In the present study, shorter hospitalization and quicker postoperative recovery were major benefits of LESSPL over MLPL. In patients with localized prostate cancer, staging LESS pelvic lymphadenectomy may be a safe alternative to conventional multiport laparoscopy.

## Background

In patients with localized high-risk prostate cancer (T1-T3, PSA ≥20 ng/ml, Gleason score ≥7) suited for radiation therapy, staging lymphadenectomy is recommended by the German Society of Radiooncology (DEGRO; http://www.degro.org). According to the S3 consensus guidelines of the DEGRO, pelvic lymph node dissection in prostate cancer patients helps to detect systemic disease and may therefore serve as therapeutic decision guidance. A minimum yield of 10 lymph nodes per patient along both external iliac arteries and the obturator fossae are recommended.

Since laparoscopic pelvic lymphadenectomy represents a surgical procedure for merely diagnostic purposes, complications should be infrequent and recuperation should be quick. In recent years, laparoendoscopic single site surgery (LESS) has evolved as an advanced laparoscopic technique. It is based upon the idea of solely employing one large umbilical port without any additional ports, thus minimizing tissue trauma. LESS was initially described in 1999 [1]. In 2011, Kaouk et al. reviewed more than 1000 urologic LESS cases that had been performed worldwide, mainly in high-volume academic institutions [2]. The application of LESS in urology encompasses a wide range of procedures, including pelvic lymphadenectomy in males with localized high-risk prostate cancer [3–7]. According to a few comparative studies, laparoendoscopic single-site surgery (LESS) may yield better postoperative outcomes compared with conventional multiport laparoscopic surgery [8–10].

In our department LESS pelvic lymphadenectomy (LESSPL) is carried out with the reusable X-Cone single port platform via the umbilicus (Karl Storz, Tuttlingen, Germany). We hypothesized that patient outcomes after LESSPL, performed with the X-Cone single port are superior to those after multiport laparoscopic pelvic lymphadenectomy (MLPL) performed via four laparoscopic trocars in the lower abdomen.

## Methods

All patient enrolled in this single-center retrospective case-control study were screened and followed by the Departments of Urology and Radiation Therapy, Charité University Hospital, Berlin, Germany. Both institutions are members of the Charité Comprehensive Cancer Center, Berlin, Germany. Using our institution’s electronic database, a total of 117 patients with localized high-risk prostate cancer, who underwent laparoscopic staging lymphadenectomy before radiation therapy were identified. Between Mai 2011 and July 2013, 20 patients underwent LESS pelvic lymphadenectomy (LESSPL), whereas 97 patients underwent conventional multiport laparoscopic pelvic lymphadenectomy (MLPL) between January 2008 and April 2013. After Mai 2011, MLPL was performed in case the experienced LESS surgeon (T.F.F.) was unavailable.

Postoperative parameters were collected by a standardized questionnaire. After screening for patient privacy issues, the questionnaire and the study were approved by the local Ethics Committee at Charité University Hospital Berlin (EA1/112/12). All patients provided written informed consent for this research study. Written informed patient consent to publish their image was obtained.

Before transfer to the recovery room, all patients were given 0.2 mg fentanyl and 2 g metamizole for early postoperative analgesia. If needed, 1 g paracetamol i.v. was given during the recovery phase. After transfer to the urology ward, metamizole was continued on patient’s request. The dosage was weight adapted with a maximum of 4 grams of metamizole per patient per day. Pain on postoperative day 1 was assessed by using the numerical analog scale (NAS) as a pain score: a value of 0 indicating no pain and a value of 10 indicating most severe pain. Full recovery of normal physical activity, defined as the ability to carry out routine daily tasks (eating, ambulating, toileting, bathing and dressing) with no need for pain medications, was evaluated on postoperative day 2. The cosmetic outcome and the incidence of complications were evaluated 6 months postoperatively.

### Surgical techniques

#### Surgical equipment and setup of the reusable X-Cone single port

**Figure 1:**
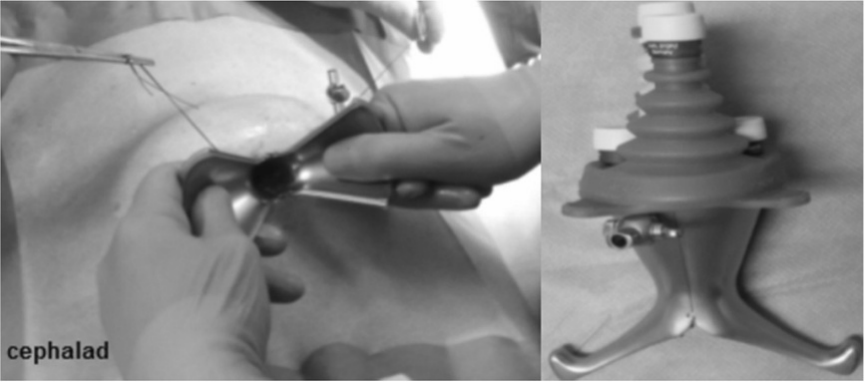
**Transumbilical introduction of the two single port half shells** **(left).** X-Cone fully assembled with a reusable silicone cap, providing 5 working channels (right).

**Figure 2:**
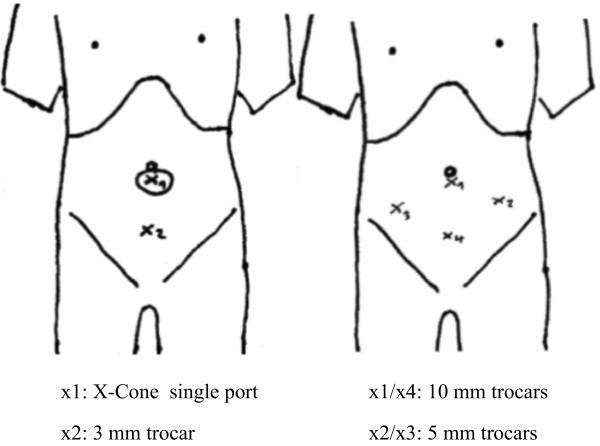
**Trocar placement in LESSPL** **(left)** **and multiport PL** **(right).**

A detailed description, of the use of the X-Cone single port platform (Karl Storz, Tuttlingen, Germany) has previously been published [10, 11]. In brief, LESSPL was performed with both surgeons at the same side of the table and the patient being placed in a supine low lithotomy position. A periumbilical incision was made to reach the abdominal space. The surgeon gently introduced the two L-shaped steel half shells of the X-Cone to form an autostatic X-shaped funnel, measuring 2.5 cm at its narrow end (Figure 1). The X-Cone was sealed with a silicone rubber cap, offering one 14 mm and four 5 mm working channels (Figure 1). From the existing armamentarium of differently shaped and prebent laparoscopic instruments (i.e. Carus, Cuschieri, Leroy) we chose the rigid Carus grasper (S-portal series, Karl Storz, Tuttlingen, Germany). It was used together with a straight laparoscopic scissors and a 5 mm extra-long 30° telescope (Karl Storz, Tuttlingen, Germany). A straight 3 mm needlescopic grasper (Karl Storz, Tuttlingen, Germany) was routinely used via an additional subumbilical trocar (Figure 2). According to the current nomenclature, adding an extra needlescopic instrument transforms LESS into needlescopic-assisted or hybrid LESS [12].

#### Surgical steps of hybrid LESS pelvic lymphadenectomy

An experienced urologic laparoscopist (T.F.F.) performed LESSPL. Towards the end of the initial learning curve, a urologic fellow performed LESSPL on one side, whenever appropriate. Cases were then completed by the experienced laparoscopist. On each side a peritoneal incision was used to expose the external iliac vessels and the obturator nerve as important anatomical landmarks. At first, lymphatic tissue along the external iliac artery was mobilized by blunt and sharp dissection. Excellent exposure of the iliac fossa is achieved by static retraction of the lower margin of the external iliac vein using the prebent Carus grasper. All 5 mm instruments including the prebent grasper were inserted transumbilically via the X-Cone. Introduction of an additional 3 mm needlescopic grasper via a subumbilical port (Figure 2) provided triangulation ofinstruments, thus improving maneuverability and safety. All lymphatic tissue along the obturator nerve was carefully dissected and removed using a 5 mm scissors and a 3 mm grasper. After retrieval of the specimen via the umbilical incision, the fascia and skin were closed using absorbable sutures.

#### Multiport laparoscopic pelvic lymphadenectomy

Fifteen urologic laparoscopists with different levels of expertise performed MLPL. The surgical steps hereof were identical to those described for LESSPL. Two 10 mm and two 5 mm trocars were placed in the lower abdomen (Figure 2). One 10 mm transumbilical trocar was used for the 0° telescope (Karl Storz, Tuttlingen, Germany). Another 10 mm trocar, placed in the midline below the umbilicus, was used for dissection and specimen extraction (Figure 2).

### Statistical analysis

SPSS 19.0 for Windows (SPSS Inc.; Chicago IL) was used for statistical analysis. For comparison of means ± standard deviation of continuous variables between the two study groups, the Student’s t-test was employed. Frequency data were compared between groups by the chi-square and Fisher’s exact test. Two-sided *P* values were reported. A *P*-value of less than .05 was considered statistically significant.

## Results

Patient characteristics are shown in Table 1. Patients in the LESSPL group tended to be older and had a significantly higher American Society of Anesthesiologists (ASA)-score, suggesting reduced physical fitness. Preoperative oncologic specifications, including the PSA value and Gleason sum, were indicative of high risk prostate cancer in both study groups. In all patients, preoperative metastatic disease was excluded with a negative bone scan.

**Table 1:** **Patient characteristics**

	LESSPL (LESS pelvic lymphadenectomy) n = 20	MLPL (Multiport pelvic lymphadenectomy) n = 97	***P***value
Patient age (mean ± SD)	71.8 (±4.2)	69.2 (±5.6)	.06
ASA-Score^a^ (mean ± SD)	2.5 (±0.5)	2.1 (±0.6)	.01*
BMI (kg/m^2^)	27.3 (±3.4)	27.7 (±4.0)	.70
Preoperative PSA value (ng/ml)	44.7 (±83.5)	32.3 (±56.2)	.50
Gleason sum	7.7 (±0.9)	7.7 (±1.0)	1.0

^a^American Society of Anesthesiologists physical status classification system.

*statistically significant.

The average operating time was significantly longer in the LESSPL group (172.1 ± 34.1 min) than in the MLPL group (116.6 ± 40.1 min) (*P* < .001) (Table 2). No conversions from LESSPL to MLPL were necessary and blood loss was minimal in all patients. The average number of retrieved lymph nodes (n =12) and the proportion of patients with positive lymph nodes (31%) was equally distributed among groups.

**Table 2:** **Postoperative outcomes**

	LESSPL (LESS pelvic lymphadenectomy) n = 20	MLPL (Multiport pelvic lymphadenectomy) n = 97	***P***value
Total operating time (min)	172.1 (±34.1)	116.6 (±40.1)	< .001*
No. retrieved lymph nodes per patient	12.5 (±5.1)	12.2 (±6.2)	.87
No. patients with positive nodes	7/20 (35%)	29/97 (30%)	.79
Hospital stay (days)	2.3 (±0.7)	3.1 (±1.2)	.01*
Pain score (NAS) postop. day 1	2.5^a^ (±2.0)	3.2^a^ (±1.7)	.12
No. patients with full recovery of normal physical activity by postop. day 2	16/19^b^ (84%)	24/75^b^ (32%)	< .001*
No. patients with complications	0/19^b^ (0%)	23/75^b^ (31%)	.01*
No. patients fully satisfied with cosmetic result	19/19^b^ (100%)	71/75^b^ (95%)	.58

^a^numerical analog scale (NAS): 0 = no pain; 10 = most severe pain.

^b^missing data: n =1 patient (LESSPL); n =22 patients (MLPL).

*statistically significant.

**Figure 3:**
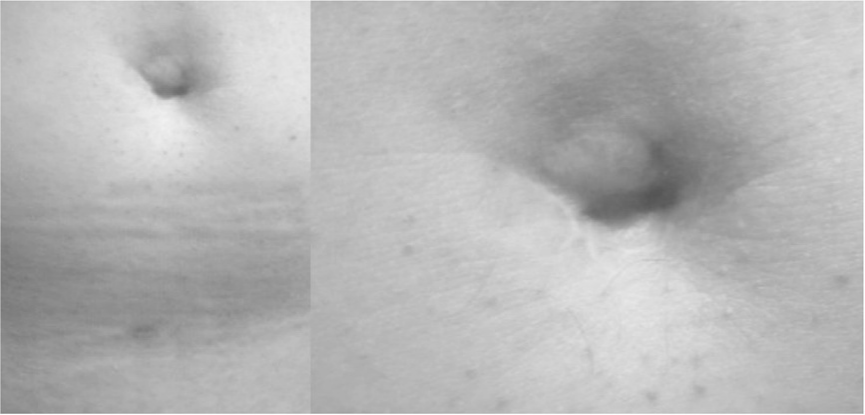
**Abdominal scars 6 months after LESSPL.**

In the LESSPL group, the length of hospital stay was significantly shorter than in the MLPL group (2.3 ± 0.7 days vs. 3.1 ± 1.2 days; *P* = .01). Pain scores on day 1, tended to be lower in the LESSPL group, but differences did not reach statistical significance: 2.5 ± 2.0 vs. 3.2 ± 1.7, respectively. By day 2, a significantly higher proportion of patients in the LESSPL group than in the MLPL group reported return to normal physical activity (84% vs. 32%; *P* < .001). All patients in the LESSPL group reported full satisfaction with the cosmetic outcome (Figure 3), whereas 95% of patients after MLPL were fully satisfied with cosmesis (*P* = .58).

No intra or postoperative complications occurred in the LESSPL group, whereas 23 out of 75 patients (31%) in the MLPL group experienced complications. In one patient intraoperative bladder injury required intracorporeal suturing and urine drainage for several days. Postoperative complications included chronic abdominal pain, reversible paresthesia of the upper thigh, hematoma, lymphoedema, wound infection (Clavien Grade 1; n =18 patients) and deep vein thrombosis (Clavien grade 2; n =1 patient). One patient with a port site hernia and 3 patients with a lymphocele required surgical intervention (Clavien grade 3; n =4 patients).

**Figure 4:**
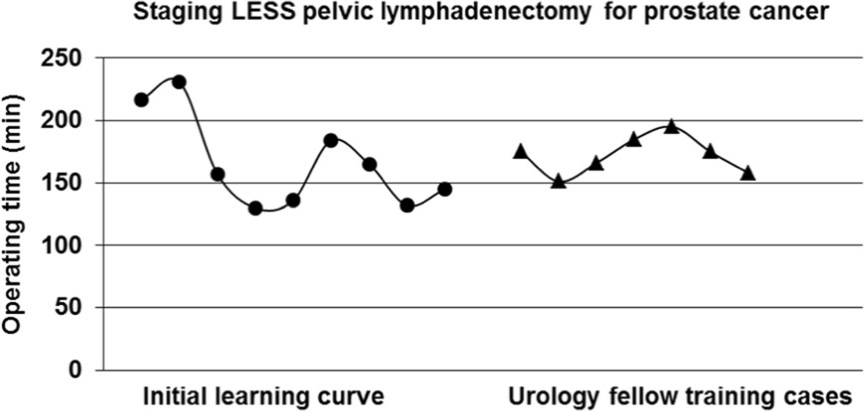
**Evolution of total operating time in 16 patients undergoing LESSPL.**

Figure 4 shows the evolution of total operating time in 16 selected patients undergoing LESSPL. Operating times are divided into two groups: the experienced laparoscopist’s initial LESSPL learning curve and subsequent teaching cases. Four patients were excluded. In one patient the surgery was stopped after a lymph node metastasis was detected upon frozen section. In 3 patients, operating times were prolonged based on technical problems, including C0_2_ leakage or malfunction of laparoscopic instruments.

## Discussion

Trocars used for conventional laparoscopy measure between 5 mm and 12 mm in diameter. The use of multiple trocars in the mid or lower abdomen may leave behind visible scars, thus compromising the cosmetic outcome. Laparoendoscopic single site surgery (LESS) typically uses the umbilicus for insertion of instruments and for specimen extraction via a larger single port. The advantage of the umbilical access is a relatively hidden and thus cosmetically favourable scar, even though the incisional length is longer than that of a conventional 12 mm trocar (Figure 3). According to the literature, umbilical hernias after single port surgery occur in less than 1% of cases [13, 14]. In our present LESSPL series, no wound complications occurred within 6 months of follow-up.

Over the past years, LESS has gained popularity in various surgical disciplines. In abdominal surgery, significant clinical benefits of LESS over conventional laparoscopy have not yet been proven [15–17]. In selected adult urologic procedures, i.e. living donor nephrectomy and varicocelectomy, LESS reduced analgesic requirements and accelerated postoperative recovery as compared to conventional laparoscopy [9, 10, 18]. In staging lymphadenectomy for endometrial cancer, outcome parameters after LESS were comparable to those after conventional multiport laparoscopy [8, 19]. One group, however, reported significantly reduced pain scores after LESS surgical staging for endometrial cancer [20].

We herein investigated potential benefits of LESS over conventional laparoscopy for surgical staging in high-risk prostate cancer patients awaiting radiation therapy. For comparison of LESS with conventional multiport laparoscopy we used a monocentric retrospective case-control study in an academic teaching environment. For the first time we showed that, apart from a significantly longer total operating time, the length of hospitalization as well as recovery of normal physical activity after pelvic lymphadenectomy was significantly shorter in the LESSPL group compared with the multiport laparoscopy group (MLPL). Moreover, the incidence of intra and postoperative complications was 0% in the LESSPL group and 30% in the MLPL group within 6 months of follow up. The average yield of pelvic lymph nodes per patient was 12.4, with no statistically significant difference between the two cohorts. In a large study on multiport laparoscopic lymph node dissection for prostate cancer, comparing the extended and modified techniques, authors reported lymph node yields of 17.8 and 9.3, respectively [21]. Given its high efficiency and low morbidity compared with the extended technique, modified laparoscopic pelvic lymphadenectomy, involving the obturator and hypogastric nodes is the preferred method for prostate cancer staging [21].

A total of 15 urologic surgeons with different levels of laparoscopic expertise performed MLPL, whereas only one experienced laparoscopist carried out LESSPL. This may contribute to the lower complication rate observed in the LESSPL group. All complications in the MLPL group were seen after discharge from hospital. It is, therefore, unlikely that longer hospitalization and slower recovery of physical activity early after MLPL may be linked to the higher complication rate in this group. Rather was reduced postoperative pain responsible for quicker recovery and shorter hospitalization in the LESSPL group. Despite a trend in favor of the LESSPL group, differences in postoperative pain scores between groups were not statistically significant. Preoperative physical fitness was assessed by the American Society of Anesthesiologists (ASA)-score. Patients in the LESSPL group showed quicker postoperative recovery, despite a significantly higher ASA-score, indicative of reduced physical fitness (Table 1).

In a recent article by Liedberg et al. on the benefits of multiport laparoscopic pelvic lymphadenectomy in prostate cancer patients, the incidence of Clavien grade 2 and 3 complications was 10.5% [22]. In comparison, the incidence of Clavien grade 2 and 3 complications in patients who underwent MLPL at our institution was 6%. In the context of the literature, our MLPL group proved to be a valid control group for the comparison with LESSPL. In our present work, no postoperative complications occurred within 6 months after LESSPL. In a series of 15 consecutive LESSPL in males with localized high risk prostate cancer, Schwentner et al. reported on one patient with inadvertent bowel injury, requiring intracorporal suturing [11]. In our view, patient safety should be a major issue in a pure diagnostic staging procedure, especially when a novel minimally invasive technique is used.

In our study, trainee involvement may have contributed to increased operative time, since MLPL and in part also LESSPL, were used to provide laparoscopic training to urology fellows. Recently, Rais-Bahrami et al. proposed that LESS training should be incorporated into the laparoscopic training program of urology residents and fellows. Establishing a credential process for LESS should be strongly considered by accrediting bodies [23].

The lack of triangulation and overcrowding of laparoscopic instruments not only accounts for a slow learning curve in almost all LESS procedures, but may also compromise patient safety. To overcome the lack of triangulation, instruments with flexible tips are increasingly used in single port surgery. In most cases, these instruments are disposable and therefore reduce cost effectiveness of LESS [9, 24]. For LESSPL, we favor a totally reusable single port platform (X-Cone, Karl Storz, Tuttlingen, Germany) with conventional straight and rigid prebent laparoscopic instruments. In our view, adding an extra 3 mm port for a needlescopic instrument improves triangulation and surgeon dexterity during LESSPL, without compromising the overall cosmetic outcome (Figure 3). Furthermore, it helps to reduce instrument overcrowding in the single port. According to the current nomenclature, “needlescopic-assisted” LESSPL performed in the present study should correctly be denoted “hybrid LESS” [12].

The majority of LESS platforms currently available on the market use disposable components. In an earlier publication, we estimated cumulative cost of 10 LESS procedures, performed with different single ports, and identified the reusable X-Cone as being the most cost-effective [10]. In their recent publication on different urologic LESS procedures, Schwentner et al. found that LESS nephrectomy performed with the X-Cone is more cost-effective than conventional multiport laparoscopic nephrectomy [11].

Limitations of our present work include the relatively small number of cases in the LESSPL group and the monocentric, retrospective design of the study. The laparoscopic procedures herein described are embedded in an academic training program. Thus, the relatively high number of participating surgeons and the inconsistency of laparoscopic expertise across study groups, i.e. one surgeon performing LESS vs. 15 surgeons performing multiport laparoscopy, represent a bias. Length of hospitalization as an outcome measure could have been biased by the fact that early discharge in the LESSPL group was probably promoted more aggressively by the medical staff than in the MLPL group. However, all patients in the LESSPL group were free to prolong their hospital stay as needed.

## Conclusions

In conclusion, our present study demonstrates that staging pelvic lymphadenectomy for prostate cancer, performed by laparoendoscopic single site surgery (LESS) is feasible and safe in the hands of an experienced urologic laparoscopist. Given its reusable components, the X-Cone single port platform is cost-effective. Compared with conventional multiport laparoscopy, LESS offers quicker postoperative recovery of prostate cancer patients awaiting radiation therapy for localized disease. Despite its limitations, our study provides the basis for prospective randomized trials to further evaluate the benefits of LESS in urology and cognate disciplines.

## Abbreviations

BMI: Body mass index
LESS: Laparoendoscopic single site
LESSPL: Laparoendoscopic single site pelvine lymphadenectomy
MLPL: Multiport laparoscopic pelvine lymphadenectomy
PL: Pelvine lymphadenectomy
PSA: Prostate specific antigene.
